# Characterizing Metabolic and Compositional Heterogeneity of Calf Muscle Using CEST MRI at 3 T

**DOI:** 10.1002/nbm.70341

**Published:** 2026-06-24

**Authors:** Huabin Zhang, Kangli Pi, Ziyan Wang, Fan Huang, Juan Wei, Patrick Liebig, Lin Chen, Yang Zhou, Yi Zhang, Varut Vardhanabhuti, Jianpan Huang

**Affiliations:** ^1^ Department of Diagnostic Radiology, Li Ka Shing Faculty of Medicine The University of Hong Kong Hong Kong China; ^2^ Medical Imaging Center, Department of Electronic Engineering and Information Science University of Science and Technology of China Hefei China; ^3^ MR Research Collaboration, Siemens Healthineers Hong Kong China; ^4^ MR Advanced Systems, Siemens Healthineers AG Erlangen Germany; ^5^ Department of Electronic Science, Fujian Provincial Key Laboratory of Plasma and Magnetic Resonance, School of Electronic Science and Engineering, National Model Microelectronics College Xiamen University Xiamen China; ^6^ Shenzhen Institutes of Advanced Technology Chinese Academy of Sciences Shenzhen China; ^7^ Zhejiang Key Laboratory of Intelligent Sensing Technology and Advanced Medical Instrument and Key Laboratory for Biomedical Engineering of Ministry of Education, College of Biomedical Engineering & Instrument Science Zhejiang University Hangzhou China; ^8^ Tam Wing Fan Neuroimaging Research Laboratory, Li Ka Shing Faculty of Medicine The University of Hong Kong Hong Kong China

**Keywords:** adiposity, calf muscle, chemical exchange saturation transfer–magnetic resonance imaging (CEST MRI), double‐step multi‐pool Lorentzian fitting (DMPLF), dual energy X‐ray absorptiometry (DXA)

## Abstract

Different human calf muscles have specialized functions, leading to inherent metabolic differences. Imaging the metabolic and compositional properties of calf muscles is important for evaluating muscle health. This study aims to characterize the metabolic and compositional heterogeneity of calf muscle by chemical exchange saturation transfer (CEST) magnetic resonance imaging (MRI) at 3 T. CEST MRI was performed on 14 healthy volunteers. CEST contrasts (amide, PCr, rNOE, and MT) were extracted from segmented muscles, and used for correlation analysis with age, fat content, BMI, and muscular strength. Distinct CEST signatures were observed between deep stabilizer and superficial powerhouse muscles. Significant correlations (e.g., rNOE vs. strength) revealed the impact of adiposity on muscle physiology. This study demonstrates the feasibility of CEST MRI for noninvasively characterizing muscle‐specific metabolic and compositional alterations, revealing distinct signatures between functionally distinct muscle groups and significant associations with adiposity and body composition.

## Graphical abstract

This study used 3 T CEST MRI to characterize metabolic and compositional heterogeneity in eight healthy calf muscles, focusing on amide, PCr, rNOE, and MT contrasts. Distinct signatures emerged between deep stabilizer and superficial powerhouse muscles. Significant correlations with age, fat content, BMI, and strength (e.g., rNOE vs. strength) revealed adiposity's impact on muscle physiology.

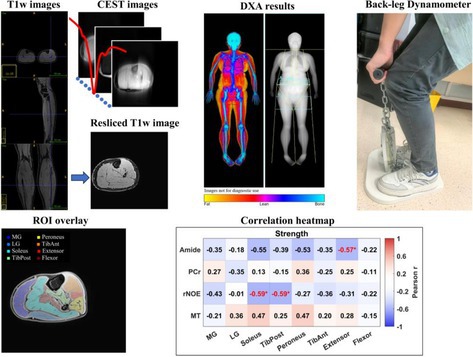

**Abbreviations**
BMI: body mass index
CEST: chemical exchange saturation transfer
Cr: creatine
DMPLF: double‐step multi‐pool Lorentzian fitting
DS: direct water saturation
DXA: dual‐energy X‐ray absorptiometry
ETL: echo train length
Extensor: extensor digitorum longus and extensor hallucis longus
Flexor: flexor digitorum longus and flexor hallucis longus
LG: lateral gastrocnemius
MG: medial gastrocnemius
MT: magnetization transfer
PCr: phosphocreatine
rNOE: relayed nuclear Overhauser effect
TibAnt: tibialis anterior
TibPost: tibialis posterior
TSE: turbo spin echo
VIBE: volumetric interpolated breath‐hold examination

## 1 Introduction

The muscles of the lower leg play a critical role in biomechanical activities demanding both explosive power and sustained endurance. The calf muscle complex, composed of muscles with diverse structural and functional characteristics, functions as an integrated unit [1, 2]. These include the gastrocnemius, which generates powerful plantar flexion, and the deeper soleus, which supports postural stability [3]. A variety of tools are currently used in muscle evaluation: electromyography detects muscle activation via electrical signals during contraction [4, 5]; back‐leg dynamometer assesses lower‐limb strength in patients with musculoskeletal injuries or hemiatrophy [6, 7]; and dual‐energy X‐ray absorptiometry (DXA) provides accurate and reproducible quantification of body composition, aiding in the diagnosis of sarcopenia [8, 9]. However, muscle quality and physical function can be influenced by factors such as age, gender, and obesity [10, 11, 12], which are often accompanied by alterations in metabolite composition [13, 14]. Consequently, there is a growing need for noninvasive imaging techniques that can capture not only structural or compositional changes but also underlying metabolic and molecular alterations in muscle.

Chemical exchange saturation transfer (CEST) magnetic resonance imaging (MRI) is an emerging molecular imaging technique that enables noninvasive detection of endogenous metabolites and macromolecules. It offers valuable insights into key physiological processes: for example, amide CEST, originating from peptides and proteins, can be used to detect muscular abnormalities in diabetic foot or to monitor pH variations in skeletal muscles [15, 16]; creatine CEST (around 2 ppm), primarily reflecting creatine (Cr) and phosphocreatine (PCr), serve as a potential biomarker for mitochondria function and energy metabolism [17, 18, 19], which is particularly relevant in conditions such as sarcopenia, disuse atrophy, or metabolic myopathy [20, 21, 22]; relayed nuclear Overhauser effect (rNOE), which is sensitive to mobile lipids and proteins [23], can provide insight into lipid–protein interactions and may serve as an early indicator of adiposity‐related metabolic remodeling [24]; and magnetization transfer (MT), originating from semisolid macromolecules [25], offers a surrogate for tissue structural integrity that can be altered in myopathies [26]. Owing to these versatile capabilities, CEST MRI holds considerable potential for probing metabolic and compositional heterogeneity within skeletal muscles [27].

This study aims to quantitatively characterize the metabolic heterogeneity of calf muscles in human using CEST MRI at clinical 3 T. We focus on two primary objectives: first, to quantitatively analyze and compare CEST contrasts (amide, PCr, rNOE, and MT) across eight distinct calf muscles; and second, to evaluate the correlations between these CEST‐derived metrics and physiological parameters, including age, body mass index (BMI), leg strength, and leg fat content. By establishing baseline CEST profiles in healthy volunteers and quantifying the magnitude of between‐muscle differences and correlations, this work provides a necessary foundation for future applications, such as patient stratification, longitudinal monitoring of disease progression, or evaluation of therapeutic interventions, where CEST metrics could serve as noninvasive metabolic biomarkers.

## 2 Methods

### 2.1 Participants

This study was approved by the Institutional Review Board of the University of Hong Kong/Hospital Authority Hong Kong West Cluster and conducted in accordance with relevant guidelines. All participants received detailed study information sheets and provided written informed consent before the MRI scans. A total of 14 healthy volunteers were recruited. MRI and DXA scans were performed at the University of Hong Kong between September and October 2024.

### 2.2 Physiological Measurements

Age, body weight, and height were recorded for each subject. BMI was calculated using the following formula:

(1) $$\mathrm{BMI}=\frac{\text{weight}\;\left(\mathrm{kg}\right)}{\text{height}\;{\left(m\right)}^{2}}.$$

**FIGURE 1:**
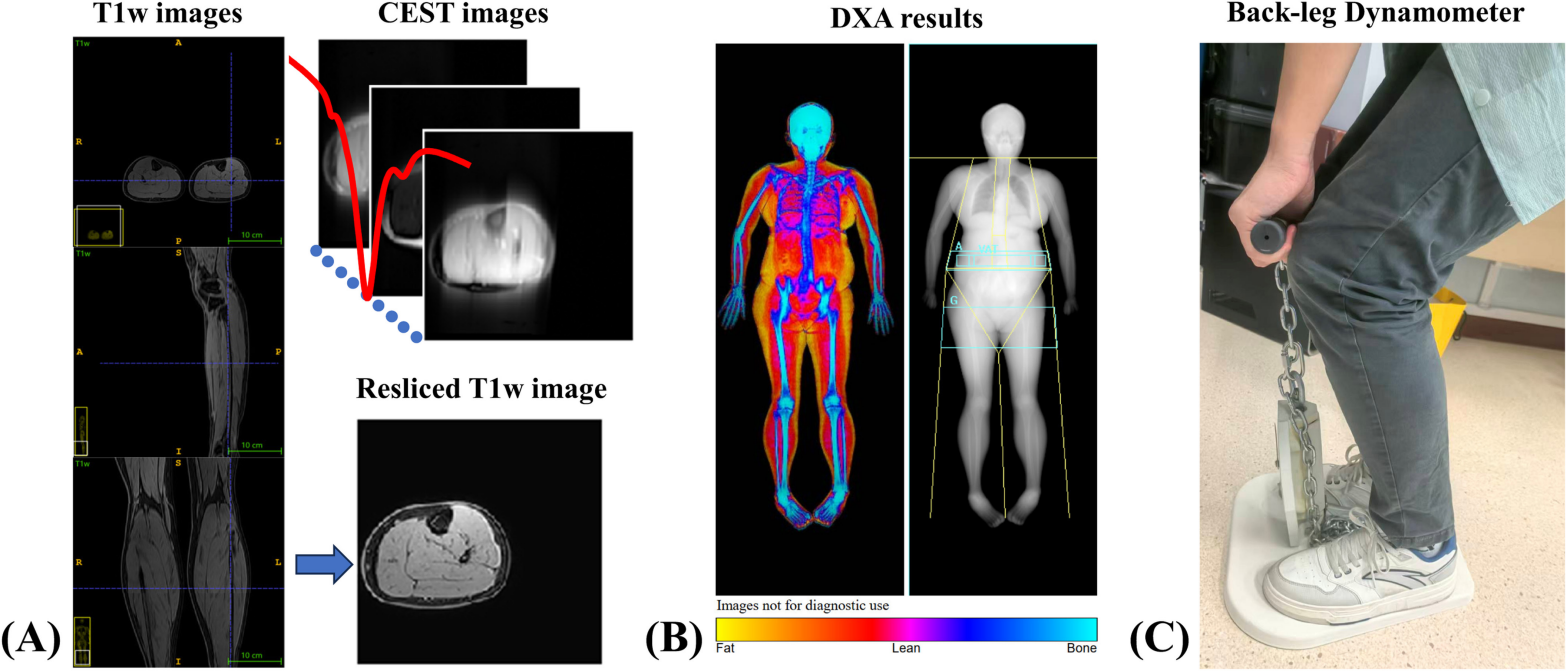
Representative data acquired in the study. (A) High‐resolution T1w anatomical image, raw CEST images, and co‐registered T1w image. (B) Body composition analysis from DXA. (C) Lower limb strength measurement using a back‐leg dynamometer. DXA, dual‐energy X‐ray absorptiometry.

Leg strength was assessed using a back‐leg dynamometer [28] within 15 to 30 min before the MRI scan. During testing, participants stood on the base with both feet, maintained an upright torso, and flexed their knees to align with the toes (Figure 1C). The chain length was adjusted so that participants could fully extend their arms to grip the handle, positioned at knee level. Participants then lifted the handle gradually in a vertical motion until no further movement was possible. The force exerted was recorded from the dial indicator. Three trials were performed per subject, and the average value was taken as the final measure of leg strength.

Left leg fat content was quantified using a Hologic Horizon A DXA full‐body fan‐beam system [29]. Participants lay supine on the scanner table, aligned so that the trunk was centered along the longitudinal axis. Palms were placed flat on the table with an air gap between the hands and torso. The feet were rotated inward, secured with Velcro straps, and positioned heels apart and toes together. Body composition analysis was conducted using the manufacturer's integrated software, which provided regional data for the left arm, right arm, trunk, left leg, right leg, subtotal region, and head (Figure 1B). The left leg fat percentage was extracted for further analysis.

Subject characteristics and physiological measurements are summarized in Table 1.

**TABLE 1:** Demographic data and subject physiological measurements.

No. of participants	14
Gender	5 females and 9 males
Age	55.2 ± 8.2 (41–69)
BMI	24.8 ± 3.2 (20.7–33.6)
Leg strength (lbs)	159.3 ± 46.7 (70–220)
Left leg fat percentage (%)	28.2 ± 6.4 (20.6–40.8)

*Note:* Value is presented as mean ± standard deviation (SD).

### 2.3 MRI Examinations

All MRI scans were conducted on a 3‐T Siemens MAGNETOM Vida scanner (Siemens Healthineers, Forchheim, Germany) to examine the left lower leg. The CEST imaging protocol employed a Gaussian pulse‐train saturation module (pulse duration = 100 ms; interpulse delay = 5 ms; number of pulses = 20; saturation duty cycle DC_sat_ = 95%; total saturation time = 2 s; root‐mean‐square saturation power B_1rms_ = 0.8 μT). The fat suppression was achieved using a spectrally selective saturation pulse centered at the dominant fat resonance frequency (−3.4 ppm relative to water) [30, 31]. One M_0_ reference image was acquired at a frequency offset of −300 ppm, along with 44 CEST images at frequency offsets ranging from −20 to 20 ppm (−20.00, −15.00, −10.00, −5.00, −4.00, −3.75, −3.50, −3.25, −3.00, −2.00, −1.00, −0.75, −0.50, −0.25, 0.00, 0.25, 0.50, 0.75, 1.00, 1.25, 1.50, 1.60, 1.70, 1.80, 1.90, 2.00, 2.10, 2.20, 2.30, 2.40, 2.50, 2.60, 2.70, 2.80, 2.90, 3.00, 3.25, 3.50, 3.75, 4.00, 5.00, 10.00, 15.00, 20.00 ppm). A single‐slice frequency‐stabilized [32, 33] turbo spin echo (TSE) sequence [34] was used as a readout module with the following parameters: echo train length (ETL) = 96; repetition time (TR) = 3000 ms; effective echo time = 7.2 ms; field of view (FOV) = 200 × 200 mm^2^; matrix size = 192 × 192; slice thickness = 5 mm; GRAPPA acceleration factor = 2. With these settings, each frequency offset was acquired within a single TR of 3 s, which included the 2 s saturation module. A total of 45 offsets (including the M_0_ reference at −300 ppm) were acquired, resulting in a net acquisition time of 135 s. Including the system's prescan (approximately 5 s), the total scan time was 2 min and 20 s. High‐resolution T1‐weighted (T1w) anatomical images were acquired using a 3D volumetric interpolated breath‐hold examination (VIBE) sequence incorporating a DIXON module. The VIBE‐DIXON sequence yields both fat‐only and water‐only images. The water‐only images, designated as T1w images, were co‐registered to the CEST reference image for anatomical localization, as illustrated in Figure 1A.

To examine B_0_ and B_1_ field inhomogeneity, a single‐slice water shift and B_1_ (WASABI) [35] sequence was employed. A continuous wave (CW) saturation module was used with a saturation time of 10 ms and a B_1_ amplitude of 3.7 μT. One M_0_ reference image was acquired at a frequency offset of −300 ppm, followed by 21 images at equally spaced offsets ranging from −2 to 2 ppm. Using the same readout settings as the CEST imaging protocol, the net acquisition time for the 22 offsets was 1 min and 6 s. Including the system prescan of about 5 s, the total scan time was 1 min and 11 s.

### 2.4 WASABI Analysis and One‐Point B1 Correction

MRI data were processed using custom scripts in MATLAB (R2024b, MathWorks, MA, USA). The acquired WASABI and CEST signal $S_{\mathrm{sat}}\left(\Delta \omega \right)$ at frequency offset Δω was first normalized by the unsaturated reference signal $S_{0}$ acquired at −300 ppm, yielding the *Z*‐spectrum:

(2) $$\begin{array}{c} Z\left(\Delta \omega \right)=\frac{S_{\mathrm{sat}}\left(\Delta \omega \right)}{S_{0}}, \end{array}$$

where $Z\left(\Delta \omega \right)$ denotes the normalized signal intensity at offset Δω, ranging between 0 and 1. The WASABI *Z*‐spectrum was then fitted pixel‐wise to the following WASABI signal model using the lsqcurvefit function in MATLAB:

(3) $$\begin{array}{c} Z\left(\Delta \omega \right)=\left|c-d\cdot \sin^{2}\left(\tan^{-1}\left(\frac{\gamma B_{1}}{\Delta \omega -\delta \omega }\right)\right)\cdot \sin^{2}\left(\frac{t_{p}}{2}\cdot \sqrt{{\left(\gamma \cdot B_{1}\right)}^{2}+{\left(\Delta \omega -\delta \omega \right)}^{2}}\right)\right|, \end{array}$$

where $\left|\cdot \right|$ denotes the absolute value operator, c and d account for relaxation effects, $B_{1}$ represents the actual saturation power, and δω denotes the water frequency shift (δ B_0_). Other known parameters include the saturation pulse duration $t_{p}$ and the frequency offset Δω. The relative $B_{1}$ (r*B*_1_) map was obtained by dividing the fitted $B_{1}$ value by the nominal saturation power (3.7 μT) used in the WASABI scan. To ensure stability and robustness of the nonlinear fitting, the MultiStart function [36] was applied with 150 random initializations for each pixel. The following parameter bounds were fixed during fitting: $B_{1}$ from 1.48 to 5.92 μT, δω from −1 to 1 ppm, c from 0.2 to 1, and d from 0.5 to 2.

The r*B*_1_ map was subsequently used for one‐point *B*_1_ correction [37, 38]. This method is based on two assumptions: the *Z*‐value is linearly related to *B*_1_, and $Z_{\mathrm{ref}}=1$ when $B_{1,\mathrm{ref}}=0$. Under these assumptions, the *Z*‐value ($Z_{\mathrm{tar}}$) at the target B_1_ ($B_{1,\mathrm{tar}}$) can be derived from the measured *Z*‐value ($Z_{\mathrm{mea}}$) at the actual *B*_1_ ($B_{1,\mathrm{mea}}$) using the following equation:

(4) $$\begin{array}{c} Z_{\mathrm{tar}}\left(B_{1,\mathrm{tar}}\right)=Z_{\mathrm{ref}}+\frac{Z_{\mathrm{mea}}-Z_{\mathrm{ref}}}{B_{1,\mathrm{mea}}-B_{1,\mathrm{ref}}}\cdot \left(B_{1,\mathrm{tar}}-B_{1,\mathrm{ref}}\right)=1+\frac{Z_{\mathrm{mea}}-1}{B_{1,\mathrm{mea}}}\cdot B_{1,\mathrm{tar}}. \end{array}$$

In this study, $B_{1,\mathrm{tar}}$ was set to 0.8 μT, and $Z_{\mathrm{tar}}$ represents the *Z*‐value after *B*_1_ correction. The actual *B*_1_ value $B_{1,\mathrm{mea}}=rB_{1}\cdot B_{1,\mathrm{tar}}$ was calculated based on the WASABI‐derived r*B*_1_ value. By applying the above equation to all offsets and pixels, *B*_1_‐corrected CEST *Z*‐spectrum data were obtained and subsequently used in the fitting analysis.

### 2.5 CEST Analysis

Peaks in the *Z*‐spectrum of CEST data correspond to the presence of exchangeable protons and were modeled as Lorentzian line‐shape form $L\left(A,\Gamma ,\Delta \omega -\delta \omega_{p}\right)$: [39]

(5) $$\begin{array}{c} L\left(A,\Gamma ,\Delta \omega -\delta \omega_{p}\right)=\frac{A\cdot \frac{\Gamma^{2}}{4}}{\frac{\Gamma^{2}}{4}+{\left(\Delta \omega -\delta \omega_{p}-\Delta \delta \right)}^{2}}, \end{array}$$

with peak amplitude A, full‐width at half‐maximum (FWHM) Γ, peak offset $\delta \omega_{p}$, and *B*_0_ inhomogeneity Δδ in ppm. Under the assumption that each pool of exchangeable protons interacts independently with water protons and neglecting inter‐pool exchange effects, the measured *Z*‐spectrum was analyzed via a linear combination of multiple Lorentzian functions.

A five‐pool double‐step multi‐pool Lorentzian fitting (DMPLF) approach [40, 41] was applied, accounting for direct water saturation (DS) at 0 ppm, PCr at 2.5 ppm [42], amide at 3.5 ppm, rNOE at −3.5 ppm, and MT at −2.5 ppm [43]:

(6) $$\begin{array}{c} Z\left(\Delta \omega \right)=1-L\left(A_{\mathrm{DS}},\Gamma_{\mathrm{DS}},\Delta \omega \right)\\ \;-L\left(A_{\text{rNOE}},\Gamma_{\text{rNOE}},\Delta \omega +3.5\right)\\ \;-L\left(A_{\mathrm{MT}},\Gamma_{\mathrm{MT}},\Delta \omega +2.5\right)\\ \;-L\left(A_{\text{amide}},\Gamma_{\text{amide}},\Delta \omega -3.5\right)\\ \;-L\left(A_{\mathrm{PCr}},\Gamma_{\mathrm{Pcr}},\Delta \omega -2.0\right). \end{array}$$

Unlike conventional multi‐pool Lorentzian fitting (MPLF) that fits all pools simultaneously, the DMPLF method employs a two‐step fitting strategy to improve parameter estimation robustness. In the first step, the background pools (DS, MT, and rNOE) were fitted using *Z*‐spectral data points outside the region of interest (ROI) for CEST pools, i.e., excluding the frequency range of 1–6 ppm. In the second step, the line shape parameters (amplitude, linewidth, and center frequency) of the background pools were fixed, and the amide and PCr peaks were fitted using the whole range of the *Z*‐spectra. This two‐step strategy reduces inter‐parameter interference and has been shown to yield more robust fitting results compared to conventional one‐step multi‐pool Lorentzian fitting [40]. The initial guesses and fitting boundaries for all pools are summarized in Table 2.

**TABLE 2:** Initial values and fitting boundaries for five‐pool DMPLF analysis of CEST data.

	Initial value	Lower bound	Upper bound
$A_{\mathrm{DS}}$	0.9	0.1	1
$\Gamma_{\mathrm{DS}}$	2	0.5	8
${\delta \omega }_{\mathrm{DS}}$	0	−1.5	1.5
$A_{\text{amide}}$	0.01	0	0.25
$\Gamma_{\text{amide}}$	2	0.5	15
${\delta \omega }_{\text{amide}}$	3.5	3.5	3.5
$A_{\text{rNOE}}$	0.1	0	0.4
$\Gamma_{\text{rNOE}}$	4	1	35
${\delta \omega }_{\text{rNOE}}$	−3.5	−3.5	−3.5
$A_{\mathrm{MT}}$	0.1	0	0.5
$\Gamma_{\mathrm{MT}}$	50	10	130
${\delta \omega }_{\mathrm{MT}}$	−2.5	−2.5	−2.5
$A_{\mathrm{PCr}}$	0.01	0	0.2
$\Gamma_{\mathrm{PCr}}$	2	0.5	15
${\delta \omega }_{\mathrm{PCr}}$	2.5	2.5	2.5

*Note:* Peak width (Γ) and peak offset (δω) are both in units of ppm.

### 2.6 Calf Muscle Delineation

**FIGURE 2:**
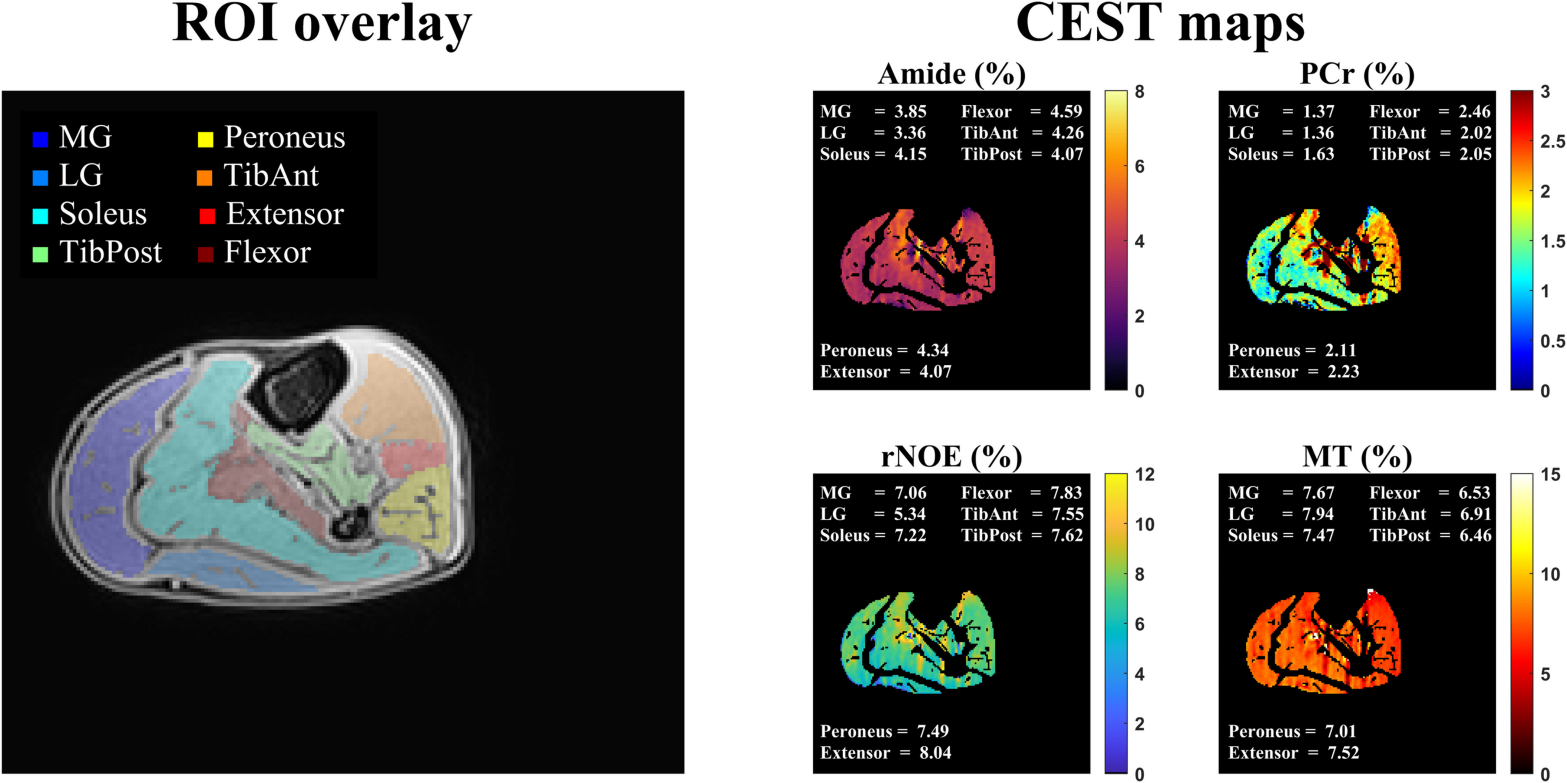
CEST maps of skeletal muscle from a representative subject. Left: eight manually delineated muscle ROIs overlaid on a T1‐weighted image. Right: corresponding CEST maps (amide, PCr, rNOE, and MT) overlaid with ROI‐averaged mean values.

Eight individual muscles of the calf were manually segmented on the co‐registered T1‐weighted images by using ITK‐SNAP software: medial gastrocnemius (MG), lateral gastrocnemius (LG), soleus, tibialis posterior (TibPost), peroneus, tibialis anterior (TibAnt), extensor digitorum/hallucis longus (Extensor), and flexor digitorum/hallucis longus (Flexor), as shown in Figure 2. The initial segmentations were performed by a doctoral researcher with a background in medical imaging, following the anatomical guidelines described in a previous study [44]. All segmentations were then reviewed and confirmed by a senior radiologist. To minimize the influence of pulsation and susceptibility artifacts, large vessels, visible tendons, and regions near bone interfaces were carefully excluded from the ROIs.

### 2.7 Regional and Statistical Analysis

Regional variations in the four CEST contrasts were compared across the eight delineated calf muscles. Statistical analyses were performed to evaluate correlations between the CEST metrics and four physiological parameters: age, left leg fat content, BMI, and leg strength. ROI‐size weighted Pearson correlation coefficients were calculated for each variable pair. Correlation analysis between the CEST metric and the physiological measurement were performed to determine statistical significance, with a *p*‐value < 0.05 considered statistically significant.

## 3 Results

### 3.1 CEST Maps of Calf Muscles

**FIGURE 3:**
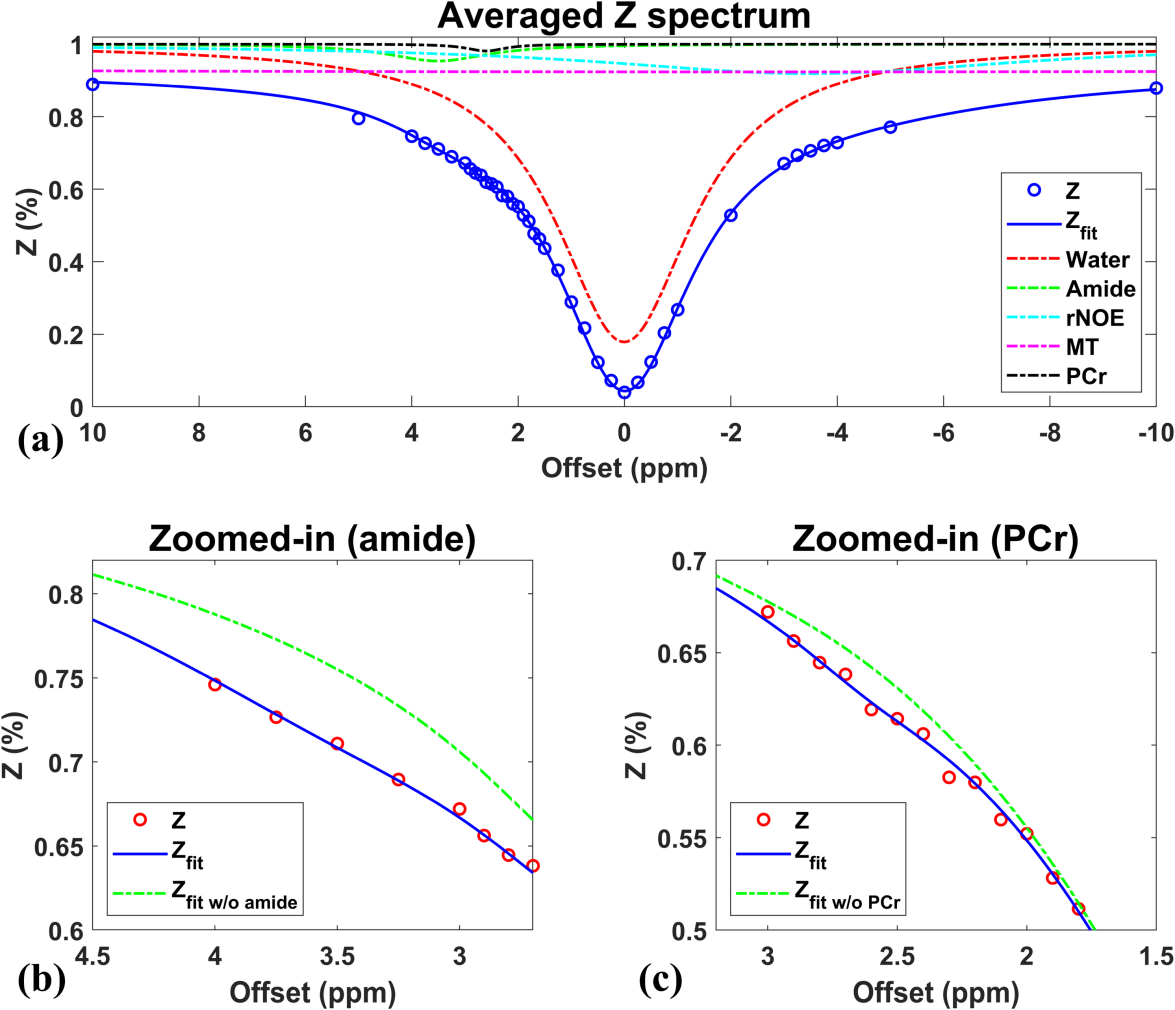
Averaged *Z*‐spectrum and Lorentzian fitting results derived from all muscle ROIs of a representative subject. (a) Full *Z*‐spectrum with five‐pool Lorentzian fitting results. (b) Zoomed‐in view of the *Z*‐spectrum around the amide pool: the blue line represents the fitted *Z*‐spectrum including all five pools, whereas the green line represents the fitted *Z*‐spectrum excluding the amide pool. (c) Zoomed‐in view of the *Z*‐spectrum around the phosphocreatine (PCr) pool: the blue line shows the fitted *Z*‐spectrum including all five pools, and the green line shows the fitted *Z*‐spectrum without the PCr pool.

Figure 2 shows CEST maps (amide, PCr, rNOE, and MT) from a representative subject along with the corresponding ROI‐averaged contrast mean values. The CEST maps of skeletal muscle reveal spatial heterogeneity in CEST contrasts across different muscles. For instance, for the rNOE and amide signals, the soleus muscle (7.22% and 4.15%, respectively) exhibits higher levels than the LG muscle (5.34% and 3.36%, respectively). Figure 3a presents details of the DMPLF fitting results, with panels Figure 3b and Figure 3c showing zoomed‐in views highlighting the CEST effects of the amide and PCr pools, respectively.

### 3.2 Distribution of CEST Contrasts Across Muscle Groups

**FIGURE 4:**
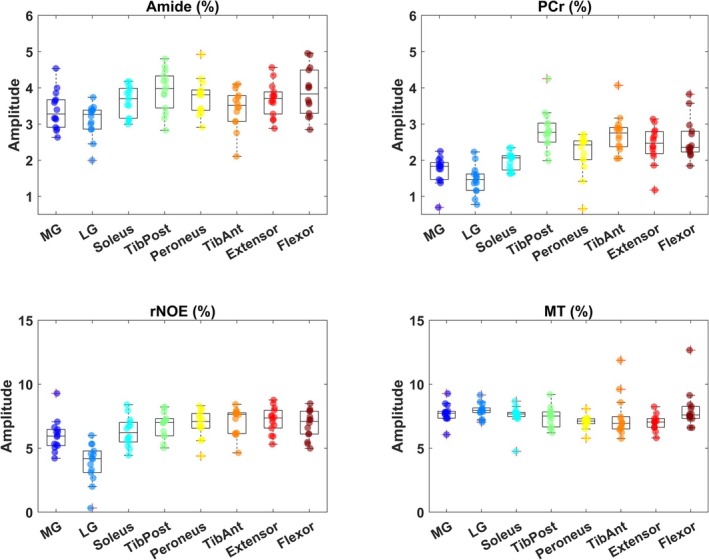
Group‐level distribution of CEST contrasts (amide, PCr, rNOE, and MT) across calf muscles. Extensor, extensor digitorum/hallucis longus; Flexor, flexor digitorum/hallucis longus; LG, lateral gastrocnemius; MG, medial gastrocnemius; MT, magnetization transfer; rNOE, relayed nuclear Overhauser effect; TibAnt, tibialis anterior; TibPost, tibialis posterior.

The distribution patterns of the four CEST contrasts across the eight examined muscles from 14 subjects are illustrated in Figure 4. The deep‐seated TibPost showed relatively high signal intensities in amide, PCr, and rNOE. In contrast, the superficial gastrocnemius muscles (MG and LG) demonstrated lower amide, PCr, and rNOE signals but higher MT signals. Among the four commonly studied calf muscles (soleus, MG, LG, TibPost), the CEST signals in the soleus were generally intermediate between those of the TibPost and the gastrocnemius muscles. Complete data for all muscles are provided in Table 3.

**TABLE 3:** Mean and SD parameters of CEST contrasts calculated from the healthy human calf muscle (*n* = 14).

Muscles	Amide (%)	PCr (%)	rNOE (%)	MT (%)
MG	3.383 ± 0.531	1.726 ± 0.385	5.973 ± 1.244	7.751 ± 0.716
LG	3.106 ± 0.457	1.461 ± 0.394	3.858 ± 1.499	7.948 ± 0.575
Soleus	3.634 ± 0.412	1.974 ± 0.250	6.286 ± 1.143	7.532 ± 0.877
TibPost	3.922 ± 0.573	2.836 ± 0.546	6.815 ± 0.988	7.420 ± 0.791
Peroneus	3.735 ± 0.510	2.177 ± 0.572	6.907 ± 1.098	7.045 ± 0.513
TibAnt	3.419 ± 0.530	2.714 ± 0.533	7.063 ± 1.056	7.461 ± 1.595
Extensor	3.672 ± 0.469	2.430 ± 0.510	7.195 ± 1.005	6.992 ± 0.598
Flexor	3.882 ± 0.662	2.568 ± 0.560	6.848 ± 1.103	8.016 ± 1.496

### 3.3 Correlation Analysis Between CEST Signals and Physiological Parameters

**FIGURE 5:**
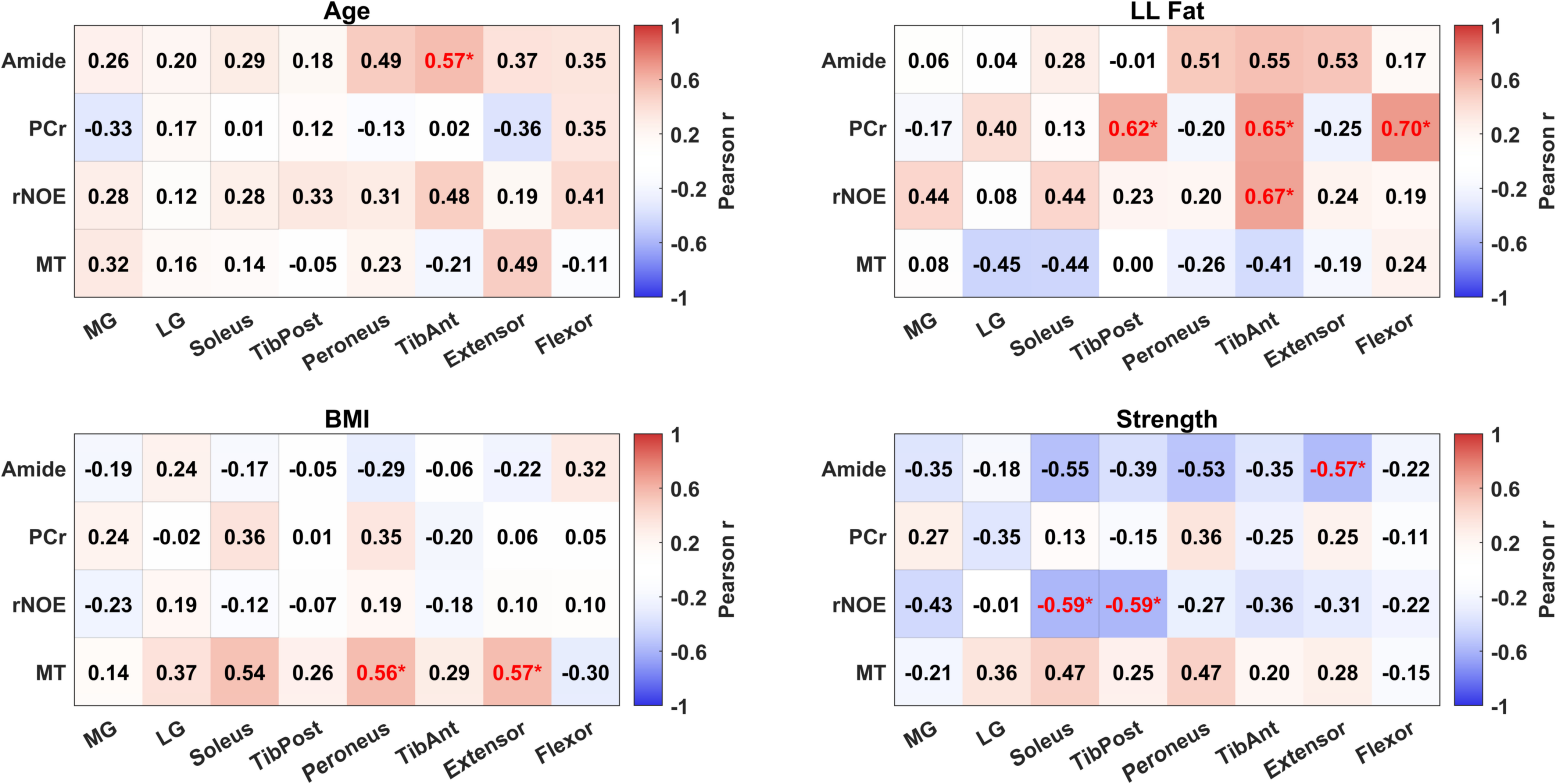
Correlation heatmap between four CEST contrasts (rows) measured in each muscle and the four physiological parameters (age, LL Fat, BMI and strength) in eight muscle ROIs (columns). Positive and negative correlations are indicated in red and blue, respectively. Asterisks and red text denote statistical significance. Extensor, extensor digitorum/hallucis longus; Flexor, flexor digitorum/hallucis longus; LL Fat, fat content of the left leg; LG, lateral gastrocnemius; MG, medial gastrocnemius; MT, magnetization transfer; rNOE, relayed nuclear Overhauser effect; TibAnt, tibialis anterior; TibPost, tibialis posterior.

**FIGURE 6:**
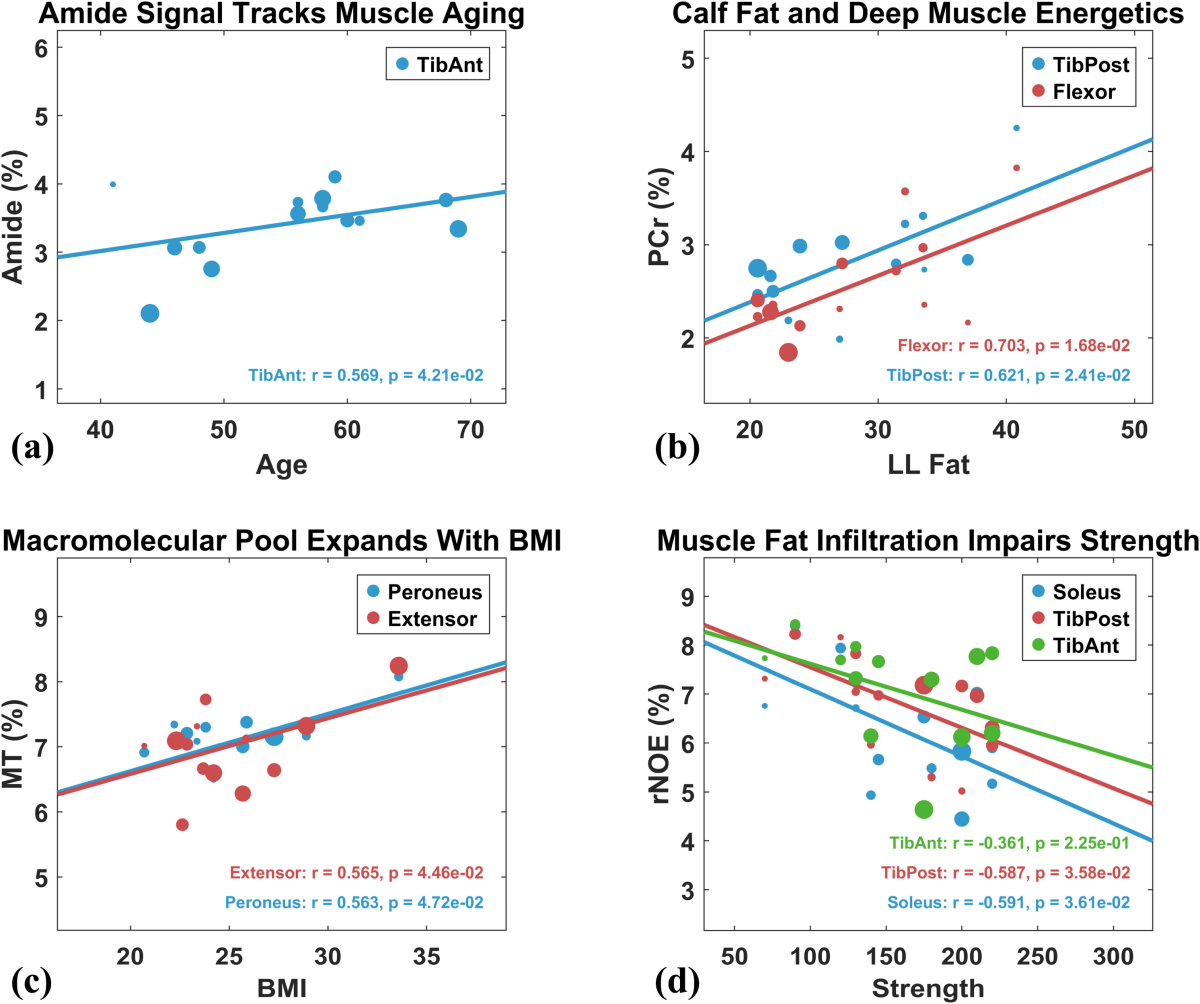
Scatter plots highlighting four specific, significant relationships: (a) Amide signal in TibAnt positively correlates with age. (b) PCr in TibPost and Flexor positively correlates with fat content. (c) MT in Peroneus and Extensor positively correlates with BMI. (d) rNOE in Soleus, TibPost, and TibAnt negatively correlates with strength. Extensor, extensor digitorum/hallucis longus; Flexor, flexor digitorum/hallucis longus; LL Fat, fat content of the left leg; MG, medial gastrocnemius; MT, magnetization transfer; PCr, phosphocreatine; rNOE, relayed nuclear Overhauser effect; TibAnt, tibialis anterior; TibPost, tibialis posterior.

The results of the correlation analysis between CEST signals and physiological parameters are summarized in Figure 5 (correlation heatmap) and Figure 6 (scatter plots depicting selected significant correlations). Specifically, Figure 6a shows a positive correlation between the amide signal in the TibAnt and age. Figure 6b presents a positive correlation between PCr in the TibPost and Flexor muscles with fat content. Figure 6c illustrates a positive correlation between MT in the Peroneus and Extensor muscles with BMI. Figure 6d demonstrates a negative correlation between rNOE in the soleus, TibPost, and TibAnt with muscle strength.

Amide signal in the TibAnt muscle was found to be significantly and positively correlated with age, which may be attributed to the aging‐related increase in collagen content [45, 46]. For BMI, the MT signal in the Peroneus and Extensor muscles showed a significant positive correlation with BMI. This may be explained by the fact that elevated BMI can alter gait patterns and foot strike biomechanics, thereby imposing greater mechanical load on muscles responsible for lateral stabilization and foot posture control. In response, these muscles may undergo adaptive remodeling with increased deposition of structural macromolecules.

Regarding left‐leg fat percentage, several significant correlations were identified:

- PCr signals in the TibPost, TibAnt, and Flexor muscles showed positive correlations with fat percentage.
- The rNOE signal in the TibAnt correlated positively with fat percentage.

With respect to leg strength, the following correlations were identified:

- The amide signal in the Extensor correlated negatively with strength.
- Furthermore, rNOE signal in the soleus and TibPost exhibited negative correlations with leg strength.

### 3.4 Influence of *B*_0_ and *B*_1_ Inhomogeneities

**FIGURE 7:**
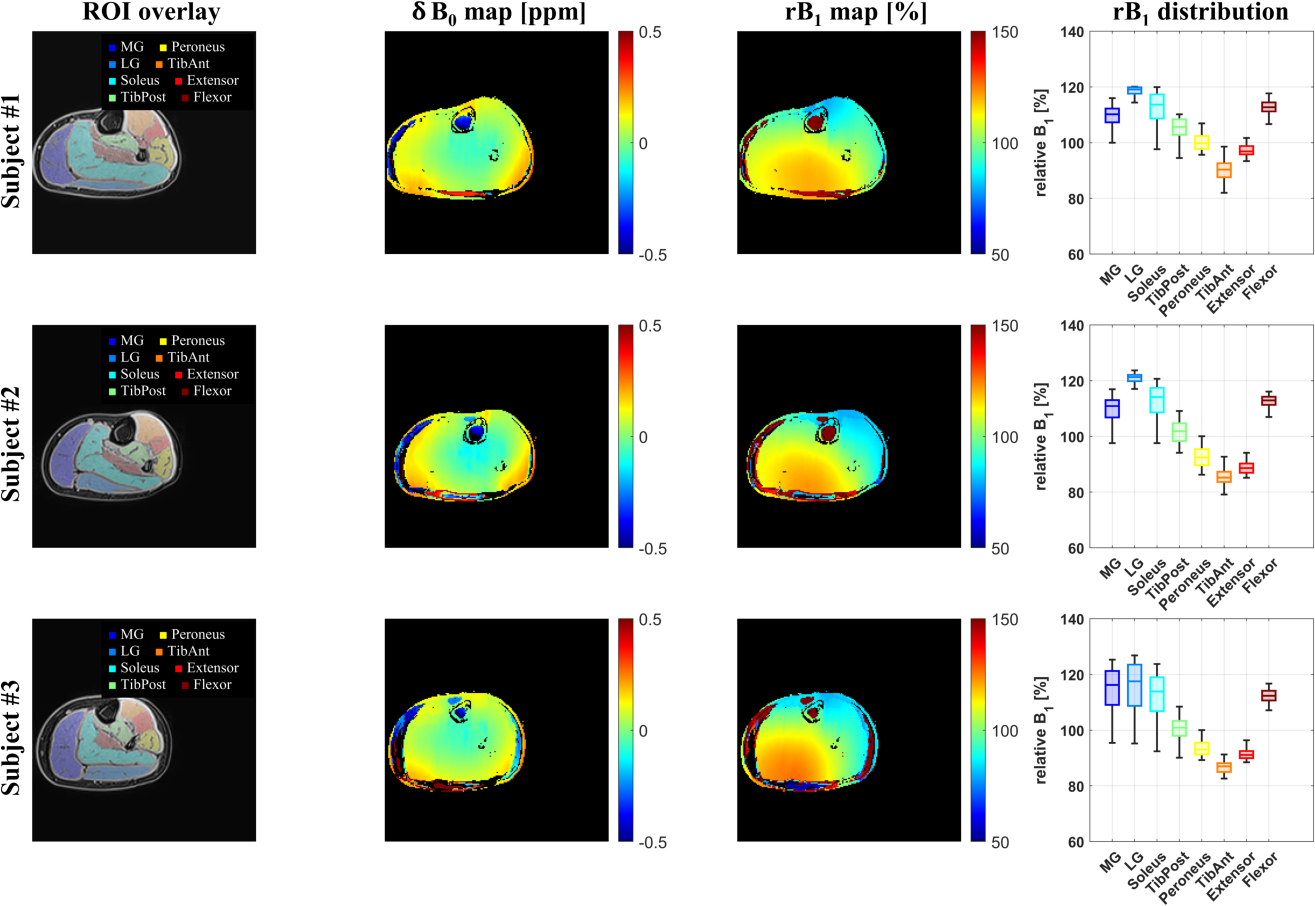
WASABI results of three subjects. Left: eight muscle ROIs. Middle: *B*_0_ field shift and relative *B*_1_ (r*B*_1_) maps calculated from WASABI. Right: r*B*_1_ distribution across all muscle ROIs. Extensor, extensor digitorum/hallucis longus; Flexor, flexor digitorum/hallucis longus; LG, lateral gastrocnemius; MG, medial gastrocnemius; MT, magnetization transfer; rNOE, relayed nuclear Overhauser effect; TibAnt, tibialis anterior; TibPost, tibialis posterior; WASABI, water shift and *B*_1_.

To explore the influence of *B*_0_ and *B*_1_ inhomogeneities, the δ*B*_0_ and r*B*_1_ maps of three subjects obtained using WASABI are presented in Figure 7. Results from all three subjects demonstrated high consistency: δ*B*_0_ values predominantly ranged between −0.2 and 0.2 ppm, while r*B*_1_ values varied from 80% to 120%. The field inhomogeneity exhibited a distinct spatial pattern. In the δ*B*_0_ maps, positive shifts were observed in the lateral regions of the calf, whereas negative shifts were detected within the interior muscle regions. For r*B*_1_, higher values were found in areas closer to the scanner bed, particularly in the gastrocnemii and soleus muscles, while lower values were noted in anterior muscle groups such as the tibialis anterior and extensor muscles.

**FIGURE 8:**
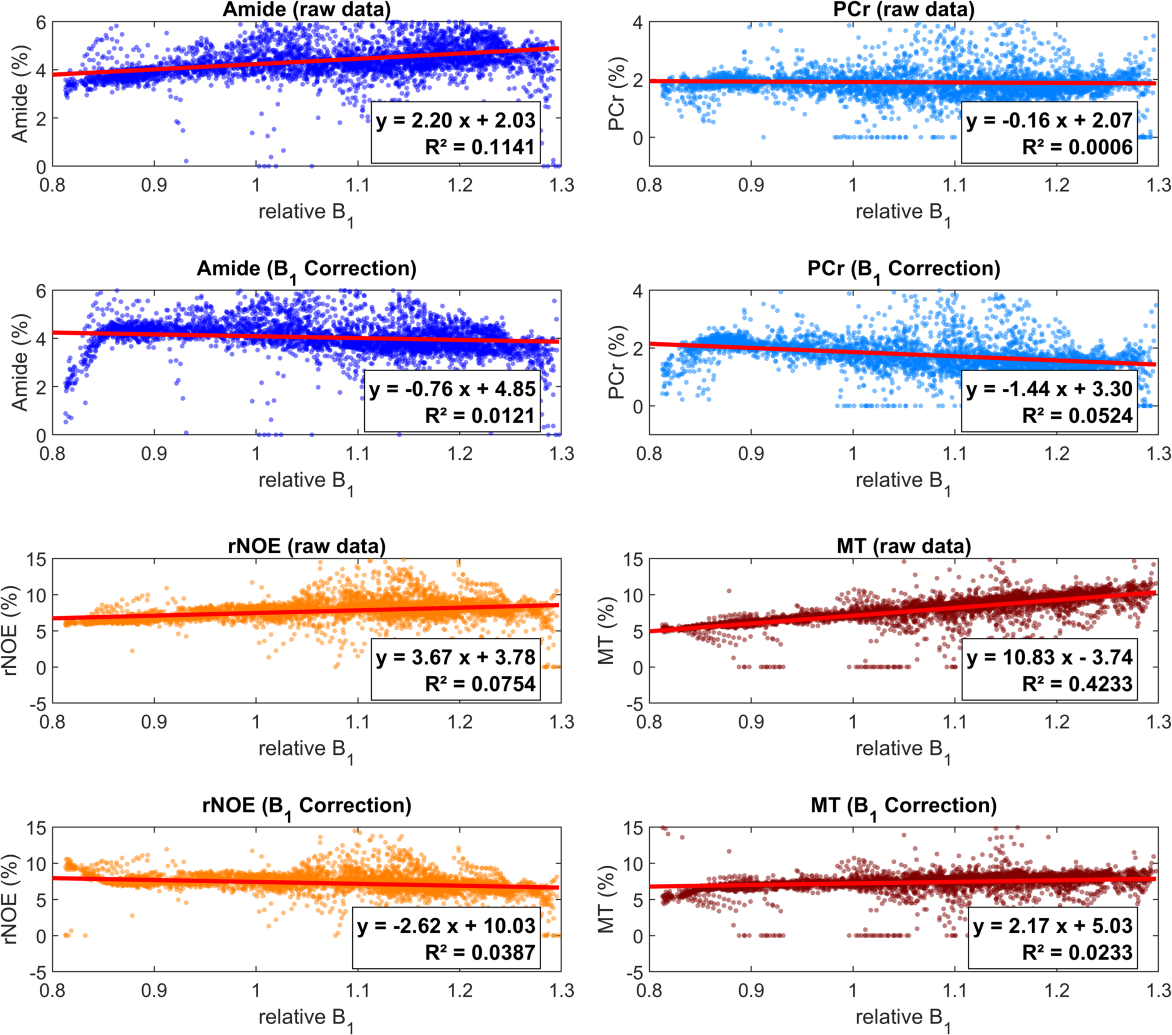
Scatter plot of CEST contrast versus relative *B*_1_ (r*B*_1_) with and without one‐point *B*_1_ correction, shown in a representative subject. The red line represents the linear fit between CEST contrast and r*B*_1_. The linear fitting equation and coefficient of determination (*R* ^2^) are displayed in the lower‐right corner. MT, magnetization transfer; rNOE, relayed nuclear Overhauser effect.

Figure 8 illustrates the effect of the one‐point *B*_1_ correction used in this study on a representative subject. Before *B*_1_ correction, both amide and MT contrast were significantly influenced by r*B*_1_, with coefficients of determination (*R* ^2^) of 0.1141 and 0.4233, respectively, from linear fitting. After applying the one‐point *B*_1_ correction, the *R* ^2^ values decreased to 0.0121 and 0.0233, respectively, indicating that the effect of spatial heterogeneity in r*B*_1_ on CEST contrast was effectively mitigated.

## 4 Discussion

This study demonstrates that CEST MRI at 3 T can effectively delineate the inherent metabolic and compositional heterogeneity of human calf muscles in vivo. The key finding is a consistent function‐dependent pattern: deep stabilizer muscles (e.g., tibialis posterior) exhibit higher amide, PCr, and rNOE signals, while superficial powerhouse muscles (e.g., gastrocnemii) show an opposite trend with higher MT effects. Furthermore, the identified correlations between specific CEST contrasts (notably rNOE and PCr) and physiological metrics of adiposity and strength provide direct molecular‐level evidence linking body composition to intramuscular physiology. Collectively, these findings demonstrate that CEST MRI is a potential noninvasive tool capable of probing muscle‐specific properties relevant to health and function.

### 4.1 Distinct CEST Profiles of the Tibialis Posterior

Our results demonstrate that the TibPost exhibited the highest signals among the muscles for amide, PCr, and rNOE contrasts (Figure 4). The elevated amide signal suggests a high concentration of mobile proteins and peptides, potentially reflecting the dense population of mitochondria and metabolic enzymes essential for the sustained, aerobic function of this postural muscle [47]. As a key muscle in maintaining the foot arch and controlling inversion, the TibPost depends on finely tuned, continuous contraction supported by abundant metabolism‐related and structural proteins. The stronger rNOE is consistent with its known predominance of slow twitch (type I) fibers, which are associated with higher mitochondrial density and intramyocellular lipid content [48].

A similar distinction was observed for PCr. Under resting conditions, Cr and PCr exist in a reversible equilibrium [49]. During high‐intensity exercise, PCr is broken down to regenerate adenosine triphosphate (ATP), with the total creatine pool (Cr + PCr) remaining constant [18]. Since the muscles were fully at rest during scanning, with Cr and PCr in dynamic equilibrium, the elevated PCr signal in the TibPost would point to a larger Cr‐PCr energy‐buffer pool, aligning with the endurance‐oriented, sustained‐activity profile of this postural muscle.

### 4.2 Characteristic CEST Profiles of Gastrocnemius

The MG and LG generally displayed lower amide, PCr, and rNOE signals compared to the TibPost and soleus (Figure 4). Notably, the LG showed the lowest rNOE across all muscles. The gastrocnemius muscles are characterized by a high proportion (approximately 50%) of fast‐twitch (Type II) fibers [50], which are primarily recruited for explosive movements such as running and jumping. The higher glycolytic capacity of fast‐twitch fibers [51] may lead to a lower intracellular pH, potentially reducing the measured amide signal by slowing the amide proton exchange rate [52]. Although these fibers possess a well‐developed sarcoplasmic reticulum, their lower mitochondrial density and distinct composition of contractile proteins may collectively contribute to the relatively weaker rNOE signal. The lower PCr signal in the MG and LG may reflect an adaptation for rapid phosphocreatine turnover rather than large‐scale storage, suiting their high‐power output demands.

### 4.3 Intermediate CEST Signature of the Soleus

The soleus consistently exhibited the second‐highest signal intensities across most CEST contrasts, positioning it intermediately between the TibPost and the gastrocnemius (Figure 4). Composed predominantly of slow‐twitch (Type I) fibers [53], the soleus is specialized for endurance activities such as maintaining upright posture [54]. Slow‐twitch fibers are characterized by higher mitochondrial content and a greater reliance on oxidative metabolism [55]. The relatively high amide and rNOE signals in the soleus are consistent with this profile, reflecting active protein synthesis and a dense macromolecular environment.

### 4.4 Positive Correlation Between Fat Percentage and PCr Signal: A Potential Compensatory Mechanism

A positive correlation was observed between leg fat percentage and PCr signals in the TibPost and Flexor muscles (Figure 6b). We speculate that this may represent a functional compensatory mechanism. As deep postural muscles essential for maintaining the foot arch and executing fine foot movements, the TibPost and Flexor may require sustained higher energy expenditure to support postural control under increased bodily load from higher fat content. This notion is supported by a previous study reporting a positive correlation between body fat percentage and energy expenditure for posture maintenance in a fasted state [56]. The increased mechanical load may upregulate the demand for readily available energy storage within these muscle fibers, potentially manifesting as an enhanced PCr signal.

### 4.5 Associations of CEST Signals With Fat Infiltration and Muscle Degradation

A negative correlation was found between leg strength and rNOE signals in the soleus, TibPost, and TibAnt (Figure 6d). Given that the rNOE signal is sensitive to the conformation and density of macromolecules such as proteins and lipids, this inverse relationship may indicate that in states like fat infiltration [57], an increase in adipose tissue within the muscle contributes more to the rNOE signal while concurrently impairing muscle quality and contraction efficiency, ultimately leading to reduced strength.

### 4.6 Impact of Field Inhomogeneity

Magnetic field inhomogeneity represents a critical factor influencing CEST quantification. *B*_0_ and *B*_1_ inhomogeneities affect the labeling coefficient and spillover factor in the CEST signal model, thereby altering the observed contrast [58]. Typically, the contribution of MT to the *Z*‐spectrum increases with *B*_1_ power [59], and different CEST contrasts exhibit distinct optimal saturation powers [60]. In this 3 T MRI study, the actual B_1_ varied by approximately 20% (relative to the nominal value of 0.8 μT), with substantial regional differences across individual muscles (Figure 6). Given that the observed differences in CEST contrast signals between muscles are on the order of only 1%–2% (Figure 2), such spatial variation in B_1_ could potentially influence the conclusion regarding the spatial heterogeneity of resting‐state muscle metabolism.

*B*_0_ shifts can be corrected by incorporating *B*_0_ inhomogeneity into the fitting model (Δδ in Equation 5) when sufficient offset data are acquired [61]. *B*_1_ correction, however, requires additional postprocessing steps. In this study, we applied a one‐point *B*_1_ correction method based on a linear assumption. The effectiveness of this approach at 3 T has been demonstrated previously [37]. As shown in Figure 7, *B*_1_ correction effectively reduces the dependence of MT contrast on the r*B*_1_. Nevertheless, it should be noted that a previous 7 T study reported that the linearity assumed by the one‐point correction may vanish when *B*_1_ exceeds 0.6 μT, potentially introducing additional quantification errors [38]. In the r*B*_1_ maps shown in Figure 7, the LG muscle adjacent to the patient table exhibits the highest r*B*_1_ levels, approaching 120%. Under a nominal *B*_1_ of 0.8 μT, this corresponds to an actual *B*_1_ close to 1 μT in that region. Meanwhile, the regional distribution in Figure 4 shows that the LG muscle has the lowest PCr and rNOE signals among all measured muscles. This observed correlation between high *B*_1_ and low PCr/rNOE signals might be a coincidental result of regional distribution, but could also be partially contributed by incomplete *B*_1_ correction. Future work could consider acquiring multiple‐*B*_1_ CEST data to enable more accurate *B*_1_ correction using methods such as spline interpolation, or alternatively, improving *B*_1_ homogeneity using dielectric pads [17].

### 4.7 Technical Limitations in CEST Acquisition

This study investigated the CEST signals in resting calf muscles and their associations with physiological parameters. A primary limitation is the restriction of CEST acquisition to a single axial slice. Considering that muscle fibers are oriented longitudinally along the calf, their morphology and composition may vary across different cross‐sections. Moreover, assessing metabolic alterations during exercise holds greater clinical relevance for evaluating muscle function. Future investigations with larger cohorts and more rigorously controlled experimental conditions are required to achieve a more comprehensive understanding of muscle physiology using CEST MRI.

Another limitation concerns image quality. Noticeable blurring artifacts were present in the raw CEST images, which could affect group‐level analyses. As shown in Figure 1A, these artifacts are visible along the phase‐encoding direction (vertical axis), likely attributable to the relatively long echo train length (ETL = 96) employed in the TSE sequence, since *T*_2_ decay can lead to image degradation [62]. The implementation of accelerated imaging techniques such as compressed sensing or parallel imaging [63, 64, 65] in future studies could improve image quality by reducing the ETL while maintaining acceptable scan times. Additionally, although vascular regions were carefully excluded during ROI delineation, prominent vertical bright streaks remain visible in the soleus region in Figure 2, partly due to arterial flow artifacts from adjacent vessels. The application of flow‐compensated gradients [66] in subsequent studies may help reduce the influence of vascular signals on muscle tissue.

The presence of adipose tissue warrants special consideration in studies involving rNOE signals. The aliphatic groups responsible for rNOE appear in the *Z*‐spectrum between −1 and −5 ppm [67], overlapping with fat resonances such as methylene (−3.4 ppm) and olefinic (−2.7 ppm) protons. In this study, we employed a spectrally selective fat saturation pulse centered at −3.4 ppm prior to each readout to suppress signal from methylene protons, the dominant fat component. However, as noted in prior work [68], conventional fat saturation may not completely eliminate fat contributions in the CEST spectrum, and residual fat signal or water‐fat phase discrepancies can occasionally produce positive peaks in the *Z*‐spectrum that mimic rNOE. To assess the potential impact in our data, we examined the DIXON fat‐only images (Figure S1), which shows that fat signal is predominantly confined to peripheral subcutaneous regions, with minimal infiltration into the calf muscles of our healthy cohort. Therefore, while residual fat effects cannot be entirely excluded, their contribution to the observed rNOE differences between deep stabilizer and superficial powerhouse muscles is expected to be minimal.

### 4.8 Choice of PCr Over Cr in the Five‐Pool Fitting Model

In this study, a five‐pool DMPLF model was applied, considering only the amide (3.5 ppm) and PCr (2.5 ppm) pools within the downfield region of the *Z*‐spectrum. The Cr pool at 2.0 ppm was not included for the following reason. A previous study by Chen et al. [42] demonstrated that under conditions of relatively high saturation power (*B*_1_ > 0.4 μT) and long saturation time (*T*_s_ > 800 ms), no distinct Cr@2 ppm peak is observable in the *Z*‐spectrum. In our protocol, a higher saturation power (*B*_1,rms_ = 0.8 μT) and long saturation time (*T*_s_ = 2 s) were used, and the zoomed‐in *Z*‐spectrum in Figure 3 shows no clearly resolvable peak at 2.0 ppm. Therefore, fitting a Lorentzian line shape for the Cr component would likely yield inaccurate results. It is worth noting that in resting human skeletal muscle, previous ^31^P MRS has reported PCr concentrations of approximately 33 millimoles per liter (mM) [69], while CEST studies at 7 T have reported Cr concentrations ranging from 24.5 to 38.32 mM [70]. Thus, the concentrations of Cr and PCr are comparable. The limited distinguishability of Cr under our experimental settings is therefore not due to low concentration but rather to differences in the exchange properties between the two pools.

Despite this assumption, the PCr‐attenuated signal obtained in this study may still contain contributions from the Cr pool. However, in resting skeletal muscle where Cr and PCr are in dynamic equilibrium, we primarily interpret the PCr signal as a surrogate for the overall Cr–PCr energy buffer pool. This allows us to explore correlations between energy‐related CEST metrics and other physiological parameters. Consequently, we believe that including potential Cr contributions does not materially affect the interpretation of our resting‐state results. In future studies involving muscle exercise or dynamic interventions, where the interconversion between PCr and Cr needs to be tracked separately, it will be necessary to disentangle their individual CEST contributions. Given that quantifying creatine is more difficult at 3 T than at higher field strengths [71, 72], more tailor‐made CEST analysis methods, such as polynomial and Lorentzian line‐shape fitting (PLOF) [73], may be required to separate PCr and Cr signals.

### 4.9 Biological Interpretation and Added Value of CEST Contrasts

Beyond the quantification challenges discussed above, it is important to clarify how CEST‐derived contrasts should be interpreted biologically and what unique information they offer relative to established MRI metrics such as the Dixon fat fraction. CEST signals do not directly report metabolite concentrations; instead, they reflect the combined effects of exchangeable proton concentration, exchange rate, and environmental factors. Consequently, CEST contrasts are best understood as functional metabolic markers that capture the dynamic interplay between molecular composition and local tissue microenvironment [74].

In this study, several CEST contrasts (notably rNOE and PCr) showed significant positive correlations with fat fraction derived from Dixon imaging. Rather than merely reflecting lipid content, these positive associations suggest that adiposity is accompanied by alterations in muscle metabolism and macromolecular organization that enhance the observed CEST signals. For instance, increased rNOE with higher fat fraction may reflect greater mobile lipid content (e.g., from intramyocellular or extramyocellular lipids) contributing to the rNOE effect, or from changes in membrane dynamics and cytosolic macromolecular crowding. Thus, while Dixon fat fraction provides a static measure of lipid content, CEST offers complementary information about the functional consequences of adiposity on muscle physiology, revealing that fat infiltration is not merely a passive replacement of tissue but is associated with active metabolic remodeling.

The present findings suggest that muscle CEST MRI at 3 T may serve as a complementary tool for applications such as monitoring metabolic dysfunction in sarcopenia or diabetic myopathy, evaluating responses to rehabilitation, or stratifying individuals based on early adiposity‐related alterations. Among the available metrics, creatine CEST offers insight into energy metabolism, rNOE reflects lipid–protein interactions, and MT reports on structural integrity. The baseline profiles established here provide a reference for future studies targeting specific muscle pathologies or interventions [75].

## 5 Conclusions

In conclusion, we employed the noninvasive CEST MRI on human calf muscle for molecular imaging and correlated the CEST findings with physiological measurement results using a clinical 3 T scanner. CEST MRI revealed distinct metabolic and compositional profiles of individual calf muscles. Significant correlations between CEST contrasts (e.g., PCr, rNOE) and physiological metrics (fat, strength, BMI) imply the impact of adiposity on muscle physiology and highlight the potential of CEST MRI for assessing and monitoring muscle health.

## Author Contributions

**Huabin Zhang:** conceptualization, methodology, investigation, formal analysis, writing – original draft, visualization. **Kangli Pi:** investigation. **Ziyan Wang:** methodology, writing – review and editing. **Fan Huang:** investigation. **Juan Wei:** methodology, resources. **Patrick Liebig:** methodology, resources. **Lin Chen:** investigation, writing – review and editing. **Yang Zhou:** investigation, writing – review and editing. **Yi Zhang:** investigation, writing – review and editing. **Varut Vardhanabhuti:** validation, writing – review and editing, funding acquisition, supervision. **Jianpan Huang:** conceptualization, methodology, investigation, formal analysis, writing – review and editing, funding acquisition, supervision, project administration.

## Funding

This work was supported by National Natural Science Foundation of China (10.13039/501100001809, 82402225), Research Grants Council, University Grants Committee (10.13039/501100002920, 27100725), University of Hong Kong (10.13039/501100003803, 109000487, 109001694, 204610401, 204610519), and Health and Medical Research Fund (10.13039/501100005847, 9202366).

## Conflicts of Interest

Two of the co‐authors (authors Juan Wei and Patrick Liebig) are full‐time employees of Siemens Healthcare. The research was conducted using a Siemens MRI system. Siemens Healthcare had no role in the study design, data collection, analysis, interpretation, or manuscript writing, except for the technical support provided by the aforementioned employees. All other authors declare no conflicts of interest.

## Supporting information


**Figure S1:** Representative VIBE‐DIXON images of three subjects. Left: water‐only images. Right: fat‐only images. VIBE, volumetric interpolated breath‐hold examination.

## Data Availability

The data that support the findings of this study are available on request from the corresponding author. The data are not publicly available due to privacy or ethical restrictions.
