# Supplementary material for: Characterizing Metabolic and Compositional Heterogeneity of Calf Muscle Using CEST MRI at 3 T
Source: NMR Biomed. 2026 Jun 24;39(8):e70341. doi: 10.1002/nbm.70341 (PMC13292845; doi:10.1002/nbm.70341)
Supplement: Supplementary file 1 — Figure S1: Representative VIBE‐DIXON images of three subjects. Left: water‐only images. Right: fat‐only images. VIBE, volumetric interpolated breath‐hold examination. [file NBM-39-e70341-s001.docx]

**Supporting Information**


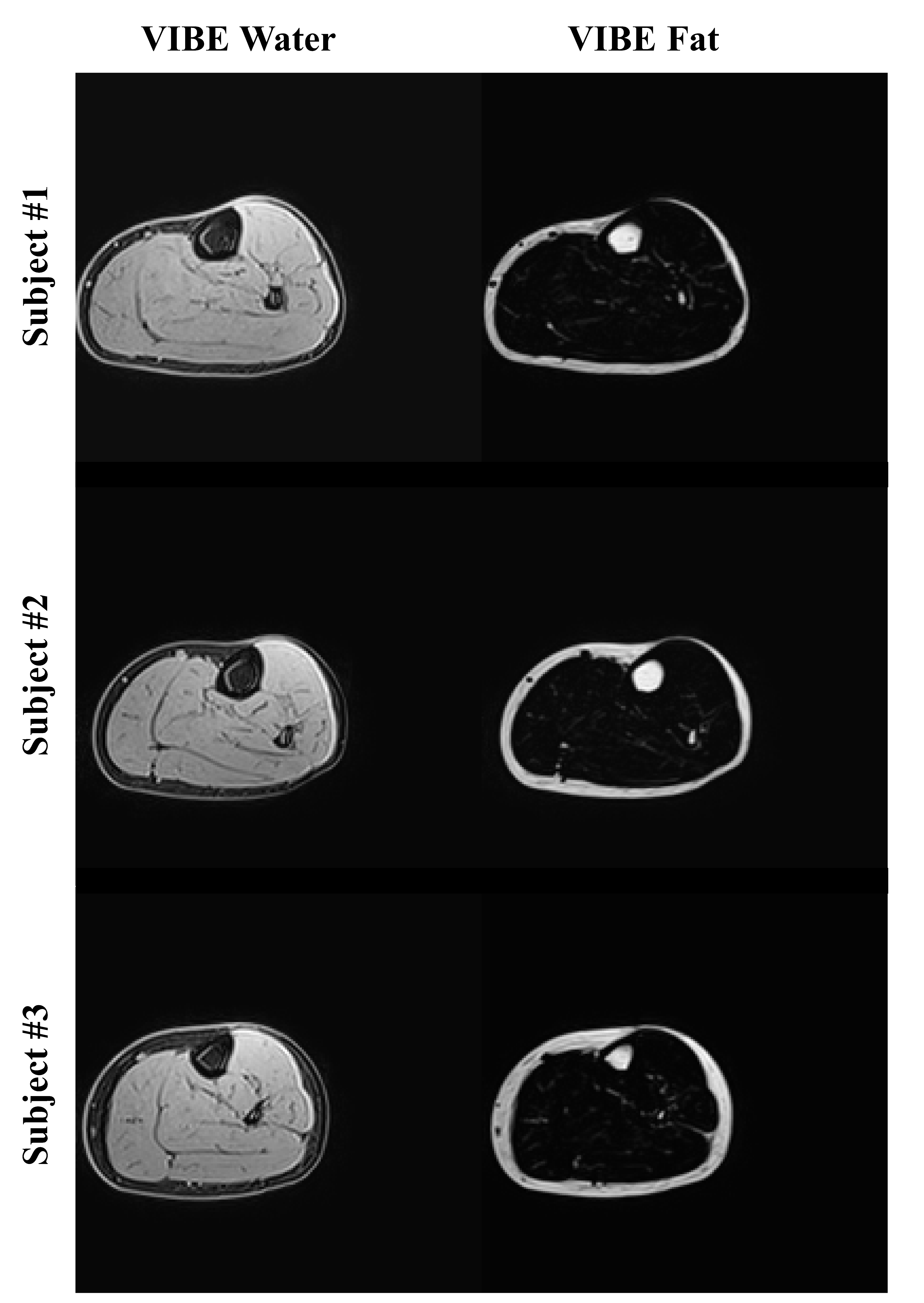


**Supporting Information Figure S1** Representative VIBE-DIXON images of three subjects. **Left:** Water-only images. **Right:** Fat-only images. VIBE, volumetric interpolated breath-hold examination.
